# Planning of kidney replacement therapy in advanced CKD using the KFRE formula in a Spanish multicenter cohort

**DOI:** 10.1093/ckj/sfag192

**Published:** 2026-06-09

**Authors:** Alba Temprado Collado, Néstor Toapanta, Mario Román Cabezas, Hector Bedoya, Efrain Tatis, Luis Gil Sacaluga, María Cristina Sánchez-Pozo, Navdeep Tangri, María José Soler, Francisco Javier Toro Prieto

**Affiliations:** Department of Nephrology, Llerena–Zafra Hospital Complex, Badajoz, Spain; Department of Nephrology, Vall d’Hebron University Hospital, Vall d’Hebron Research Institute (VHIR), Autonomous University of Barcelona, Passeig de la Vall d’Hebron 119-129, Barcelona, Spain; Department of Clinical Biochemistry, Virgen del Rocío University Hospital, IBiS/CSIC, University of Seville, Avenida Manuel Siurot s/n, Seville, Spain; Department of Nephrology, Vall d’Hebron University Hospital, Vall d’Hebron Research Institute (VHIR), Autonomous University of Barcelona, Passeig de la Vall d’Hebron 119-129, Barcelona, Spain; Department of Nephrology, Vall d’Hebron University Hospital, Vall d’Hebron Research Institute (VHIR), Autonomous University of Barcelona, Passeig de la Vall d’Hebron 119-129, Barcelona, Spain; Department of Nephrology, Virgen del Rocío University Hospital, IBiS/CSIC, University of Seville, Avenida Manuel Siurot s/n, Seville, Spain; Department of Clinical Biochemistry, Virgen del Rocío University Hospital, IBiS/CSIC, University of Seville, Avenida Manuel Siurot s/n, Seville, Spain; Department of Internal Medicine, University of Manitoba, Winnipeg, Manitoba, Canada; Chronic Disease Innovation Centre, Seven Oaks Hospital, Winnipeg, Manitoba, Canada; Department of Nephrology, Vall d’Hebron University Hospital, Vall d’Hebron Research Institute (VHIR), Autonomous University of Barcelona, Passeig de la Vall d’Hebron 119-129, Barcelona, Spain; Department of Nephrology, Virgen del Rocío University Hospital, IBiS/CSIC, University of Seville, Avenida Manuel Siurot s/n, Seville, Spain

**Keywords:** chronic kidney disease, competing risks, Kidney Failure Risk Equation, kidney replacement therapy, risk stratification

## Abstract

**Background:**

The Kidney Failure Risk Equation (KFRE) is widely used to estimate the risk of kidney replacement therapy (KRT), but evidence in real-world multicenter advanced chronic kidney disease (ACKD) cohorts—particularly among older patients with substantial competing mortality—remains limited.

**Methods:**

We conducted a multicenter retrospective cohort study including adults with ACKD [baseline estimated glomerular filtration rate (eGFR) <20 ml/min/1.73 m^2^] followed at specialized multidisciplinary clinics in Barcelona and Seville (2017–2022). The 2-year 4-variable KFRE (age, sex, eGFR, and urine albumin-to-creatinine ratio) was externally validated for predicting KRT initiation, treating death as a competing event. Discrimination was assessed using time-dependent area under the curve (AUC); calibration was evaluated using decile-based plots and quantitative metrics [calibration-in-the-large (CITL) and Brier score] using Fine–Gray competing risk methods. Clinical utility was explored using decision curve analysis (DCA) and a 20% KFRE threshold in patients aged ≥65 years.

**Results:**

A total of 503 patients were included. During follow-up, 248 patients initiated KRT and 94 died before KRT. The 2-year 4-variable KFRE showed good discrimination [time-dependent AUC 0.82, 95% confidence interval (CI) 0.73–0.91], preserved across age strata. Calibration demonstrated appropriate risk ordering across deciles with mild underestimation (CITL −0.21 by Fine–Gray; Brier score 0.23). In patients aged ≥65 years, a KFRE ≥20% was strongly associated with KRT initiation in competing risk models (subdistribution hazard ratio 5.92, 95% CI: 4.08–8.60). DCA showed positive net benefit across clinically relevant thresholds.

**Conclusions:**

In a multicenter Spanish ACKD cohort, the 2-year KFRE demonstrated robust discrimination and acceptable calibration. These findings highlight the clinical utility of the KFRE for risk stratification and KRT planning in older patients with ACKD.

## Graphical Abstract

**Graphical Abstract:**
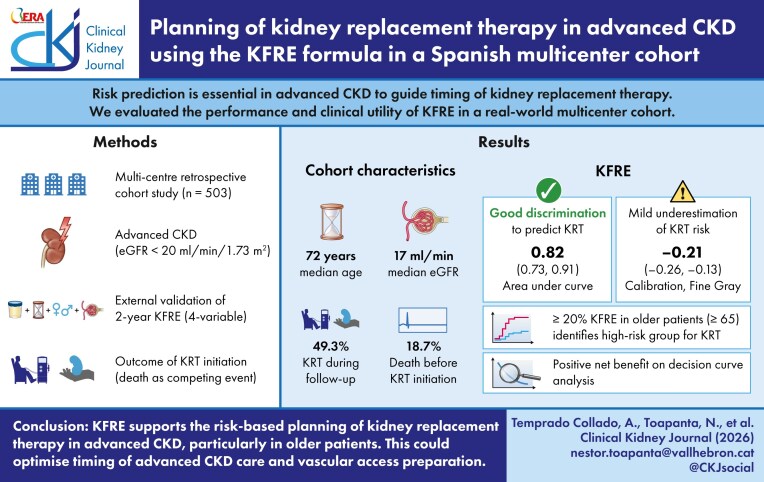

KEY LEARNING POINTS **What was known:**

- The Kidney Failure Risk Equation (KFRE) is a widely validated tool for predicting progression to kidney failure in patients with chronic kidney disease using routinely available clinical variables.
- Despite strong performance in international cohorts, evidence regarding its performance in real-world advanced chronic kidney disease (ACKD) populations, particularly in Southern Europe and among older patients, remains limited.
- In ACKD, high competing mortality may affect risk prediction and clinical interpretation, highlighting the need for validation studies that account for competing risks in nephrology care settings.

**This study adds:**

- In a real-world multicenter Spanish ACKD cohort, the 2-year 4-variable KFRE demonstrated good discrimination (area under the curve 0.82) and acceptable calibration for predicting kidney replacement therapy initiation.
- The model maintained robust predictive performance across age groups, including patients aged ≥65 years, despite the presence of competing mortality.
- KFRE-based risk stratification identified patients at substantially higher short-term risk of kidney replacement therapy, supporting its use in clinical risk assessment.

**Potential impact:**

- KFRE may support risk-based planning of kidney replacement therapy in patients with ACKD followed in specialized nephrology clinics.
- Integration of KFRE into routine clinical practice could help guide referral, monitoring intensity, and timely preparation for dialysis access or transplantation.
- Risk-based approaches may improve shared decision-making and optimize resource allocation in ACKD care.

## INTRODUCTION

Chronic kidney disease (CKD) represents a major and growing public health challenge. The *Global Burden of Disease* Study recently estimated that CKD affects >850 million adults worldwide and currently ranks among the 10 leading causes of death, with projections placing it as the fifth leading cause of global mortality in the coming decades. This increasing burden reflects not only progression to kidney failure but also a substantial rise in cardiovascular morbidity and mortality across all stages of CKD [1, 2].

Clinical decision-making is particularly complex in patients with advanced CKD (ACKD). In this population, progression is highly heterogeneous, with some patients experiencing rapid decline while others remain stable for prolonged periods. Death and cardiovascular events frequently occur before progression to kidney replacement therapy (KRT), acting as dominant competing risks and limiting the clinical usefulness of strategies based solely on estimated glomerular filtration rate (eGFR) [3, 4].

This issue is especially relevant in older patients, in whom mortality may exceed progression to kidney failure. Accurate identification of patients at high short-term risk of kidney failure is therefore essential to support timely referral to ACKD care, shared decision-making, planning of KRT, including vascular access, pretransplant assessment, and consideration of conservative kidney management when appropriate [5].

In this context, the Kidney Failure Risk Equation (KFRE) has emerged as one of the most widely validated tools for predicting progression to kidney failure. The four-variable KFRE, incorporating age, sex, eGFR, and urine albumin-to-creatinine ratio (UACR), allows estimation of the 2- and 5-year absolute risk of KRT initiation using routinely available clinical data [6]. Large multinational validation studies have demonstrated consistently high discriminative performance of the KFRE across diverse populations, although calibration has shown variability depending on baseline risk and healthcare setting [7]. Consequently, local validation remains essential before clinical implementation in specific populations.

The 2024 Kidney Disease Improving Global Outcomes (KDIGO) Clinical Practice Guideline for the Evaluation and Management of CKD recommends the use of validated risk prediction equations, such as the KFRE, to guide referral to nephrology and planning of ACKD care, emphasizing the importance of accounting for competing risks, local calibration, and individual clinical context [8]. Although the KFRE has been validated in Spanish cohorts with ACKD [9], evidence regarding its performance and clinical utility in real-world multicenter ACKD populations, particularly among older individuals with high competing mortality risk, remains limited.

The aim of this study was to externally validate the four-variable KFRE for predicting initiation of KRT in a real-world Spanish cohort of patients with ACKD, accounting for competing risk of death. Furthermore, we assessed its clinical applicability for KRT and vascular access planning, and to explore age-related differences in predictive performance using competing risk approaches.

## MATERIALS AND METHODS

### Study design and population

We conducted a multicenter retrospective observational cohort study including adult patients followed at specialized multidisciplinary ACKD outpatient clinics at Vall d’Hebron University Hospital (Barcelona, Spain) and Virgen del Rocío University Hospital (Seville, Spain). Consecutive patients initiating follow-up in ACKD clinics between January 2017 and December 2022 were screened for inclusion.

A total of 503 patients were included in the study, of whom 139 were recruited at Vall d’Hebron University Hospital and 364 at Virgen del Rocío University Hospital.

Eligible participants were aged ≥18 years, had an eGFR <20 ml/min/1.73 m^2^ at the baseline ACKD visit, were not receiving KRT at baseline, and had sufficient clinical and laboratory data available to calculate the four-variable KFRE.

Patients receiving KRT at baseline, those with missing data required for KFRE calculation (eGFR or UACR), and patients with <3 months of follow-up were excluded to ensure adequate outcome assessment.

All included patients were followed from the baseline ACKD visit until initiation of KRT, death, or completion of 2 years of follow-up, whichever occurred first.

### Variables and risk prediction models

Baseline demographic and clinical variables were extracted from electronic medical records, including age, sex, body mass index, comorbidities (hypertension, diabetes mellitus, and cardiovascular or cerebrovascular disease), and aetiology of CKD. Laboratory parameters collected at the first ACKD visit included serum creatinine, eGFR (calculated using the Chronic Kidney Disease Epidemiology Collaboration equation), and UACR. Serum creatinine and eGFR were also recorded at the time of KRT initiation, death, or end of follow-up in order to characterize kidney function at the time of the observed clinical event and to compare predicted versus observed outcomes in this retrospective cohort.

The 2-year risk of kidney failure was estimated using the four-variable KFRE, incorporating age, sex, eGFR, and UACR. KFRE estimates were calculated as probabilities (range 0–1) and percentage (0%–100%) formats. Probability estimates were used for discrimination analyses, calibration assessment, and decision curve analysis (DCA).

### Outcomes and follow-up

The primary outcome was initiation of KRT, defined as hemodialysis, peritoneal dialysis, or pre-emptive kidney transplantation. Death without prior initiation of KRT was treated as a competing event. Patients who neither initiated KRT nor died during follow-up were censored at the end of the 2-year observation period.

Time to event was defined as the number of days from the baseline ACKD visit to initiation of KRT, death, or censoring.

### Sample size calculation

The sample size was calculated using the Hanley and McNeil formula, assuming an expected area under the curve (AUC) of ∼0.85–0.90, a 95% confidence interval (CI), a maximum error of ±0.05, and a 2-year KRT event rate of 20%. The resulting minimum sample size was 517 patients [10]. Although the calculated minimum sample size was 517 patients, the final cohort comprised 503 individuals, reflecting the available eligible population during the study period.

### Statistical analysis

Baseline characteristics were summarized using standard descriptive methods; continuous variables were expressed as mean (±standard deviation) or median (interquartile range), as appropriate; categorical variables as counts and percentages. Discrimination of the KFRE was assessed using time-dependent AUC at a 2-year event horizon, estimated via nonparametric receiver operating characteristic (ROC) regression with 1000 bootstrap replications using the *cumulative_dynamic_auc* method from the *scikit-survival* library (Python 3.10.18). Cumulative incidence function curves and subdistribution hazard ratios (SHRs) were estimated using Kaplan–Meier and Fine–Gray competing risk regression models, implemented in R with the *cmprsk* and survival packages, and are reported with 95% CIs [11]. Calibration was assessed using the Brier score and calibration-in-the-large (CITL), alongside calibration plots by predicted risk deciles. DCA was conducted to assess the clinical net benefit of the KFRE at various risk thresholds [12].

### Subgroup analysis

A subgroup analysis according to age (≥65 vs <65 years) was conducted, considering the increased risk of competing events in older patients, to assess the clinical applicability of the model in this population. Statistical differences between subgroups were assessed using Mann–Whitney’s *U*-test, using Bonferroni’s correction for multiple comparisons.

### Ethics

The study was conducted in accordance with the Declaration of Helsinki, with all patient data fully anonymized prior to analysis. The study protocol was approved by the relevant institutional review boards: the Provincial Medicine Research Ethics Committee of Seville at Hospital Universitario Virgen del Rocío (Ethical Committee code: SICEIA-2025-000479, 25 February 2025) and the Institutional Review Board of Vall d’Hebron University Hospital (Protocol code: PR[AG]123/2025).

## RESULTS

### Baseline characteristics and comparison by KRT initiation status

A total of 503 patients with ACKD were included in the study, of whom 364 were recruited in Seville and 139 in Barcelona. Baseline demographic, clinical, and laboratory characteristics of the study population, stratified by center, are summarized in Table 1.

**Table 1:** Baseline characteristics of the study population by center. Data are presented as median (interquartile range) for continuous variables and number (percentage) for categorical variables. *P*-values correspond to comparisons between centers (Seville vs Barcelona) using the Mann–Whitney *U*-test for continuous variables and the χ^2^ or Fisher’s exact test for categorical variables, with Bonferroni correction for multiple comparisons.

Characteristics	Combined(*n* = 503)	Virgen del Rocío Hospital, Seville (*n* = 364)	Vall d’Hebron Hospital, Barcelona (*n* = 139)	*P*-value (adj)
Sociodemographic parameters				
Age, years	72.0 (61.5–79.0)	71.00 (61.0–79.0)	72.9 (63.7–79.1)	.999
Male sex, *n* (%)	278 (55.3%)	199 (54.7%)	79 (56.8%)	.564
Caucasian, *n* (%)	486 (96.6%)	362 (99.5%)	124 (89.2%)	**<.001**
Physical parameters				
Body mass index, Kg/m^2^	29.04 (25.40–33.27)	29.42 (26.09–33.45)	26.88 (24.56–31.68)	**<.001**
Clinical parameters				
ACR, mg/g	767.00 (140.25–2004.14)	698.00 (104.00–1950.00)	926.00 (268.00–2108.50)	.084
Initial serum creatinine, mg/dl	3.25 (2.80–3.87)	3.18 (2.73–3.80)	3.47 (3.08–4.06)	**<.001**
Initial eGFR CKD–EPI, ml/min/1.73 m^2^	17.00 (14.00–19.35)	17.40 (14.57–19.90)	15.00 (13.00–18.00)	.307
Final serum creatinine, mg/dl^a^	4.50 (3.21–5.80)	4.53 (3.00–5.91)	4.43 (3.54–5.40)	.999
Final eGFR CKD–EPI, ml/min/1.73 m^2^ ^a^	11.00 (8.00–15.00)	11.00 (8.00–16.00)	11.00 (9.00–13.50)	.999
CKD etiology, *n* (%)				
Diabetes	135 (26.8%)	104 (28.6%)	31 (22.3%)	.999
Vascular	120 (23.9%)	86 (23.6%)	34 (24.5%)	.999
Unknown	108 (21.5%)	71 (19.5%)	37 (26.6%)	.999
Glomerular	48 (9.5%)	38 (10.4%)	10 (7.2%)	.999
Tubulointerstitial nephritis	44 (8.7%)	34 (9.3%)	10 (7.2%)	.999
Polycystic kidney disease	24 (4.8%)	13 (3.6%)	11 (7.9%)	.999
Others	23 (4.6%)	17 (4.7%)	6 (4.3%)	.999
Comorbidities, *n* (%)				
Diabetes mellitus	274 (54.5%)	200 (54.9%)	74 (53.2%)	.999
Hypertension	468 (93.0%)	338 (92.9%)	130 (93.5%)	.999
Cardiovascular disease	102 (20.3%)	68 (18.7%)	34 (24.5%)	.999
Cerebrovascular disease	74 (14.7%)	60 (16.5%)	14 (10.1%)	.999
Outcomes during follow-up				
KRT events	248 (49.3%)	134 (36.81%)	114 (82.01%)	**<.001**
Death before KRT (competing event)	94 (18.7%)	88 (24.2%)	6 (4.3%)	**<.001**

^a^Final values correspond to the last available measurement before KRT initiation, death, or end of follow-up.

CKD–EPI, Chronic Kidney Disease Epidemiology Collaboration; ACR, albumin-to-creatinine ratio.

The median age of the overall cohort was 72 years, and 55.3% were male. At baseline, kidney function was markedly impaired, with a median eGFR of 17.0 ml/min/1.73 m^2^. Albuminuria was severe, with a median UACR of 767 mg/g, consistent with ACKD. Diabetic and vascular kidney disease were the most frequent underlying aetiologies, followed by kidney disease of unknown or unspecified cause. During follow-up, 248 patients (49.3%) initiated KRT, while 94 patients (18.7%) died before KRT initiation.

### Discriminative performance of the KFRE model at 2 years

**Figure 1:**
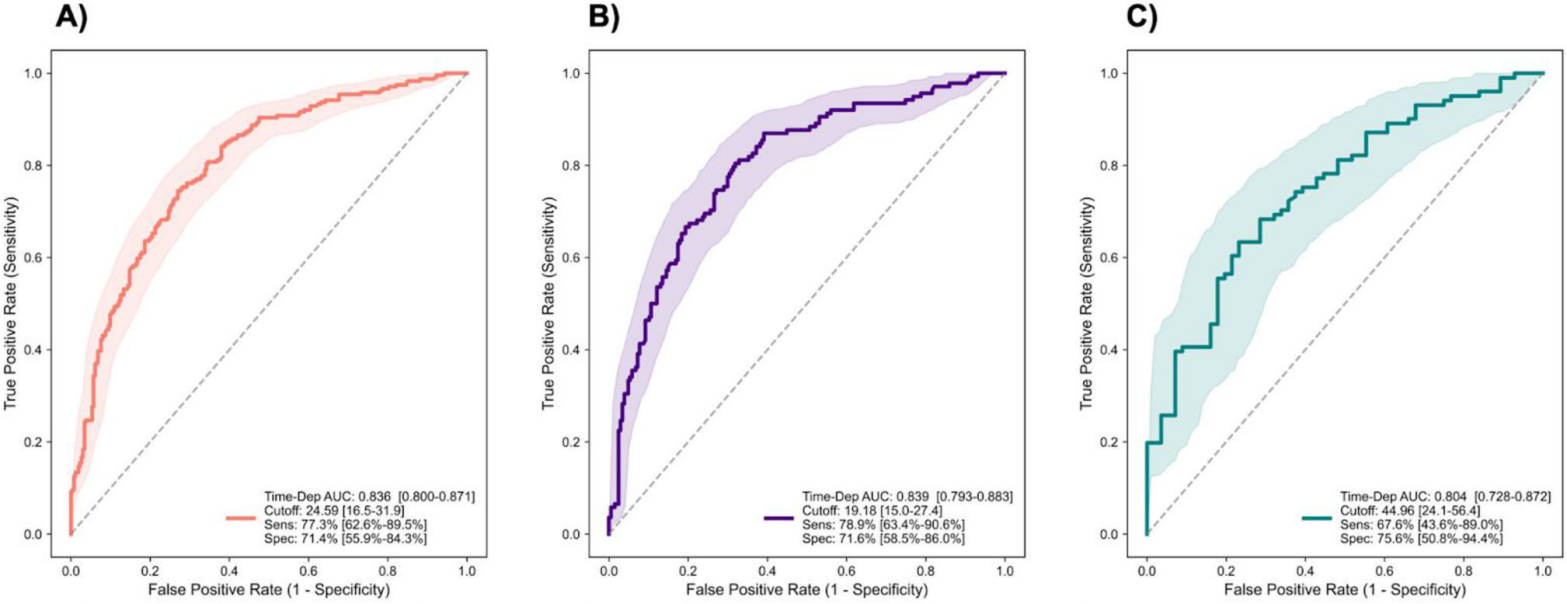
Time-dependent ROC curve (2-year horizon) for the 4-variable 2-year KFRE model. (A) Combined cohort. (B) ≥65 year. (C) <65 year.

The discriminative performance of the KFRE for predicting KRT initiation at 2 years was assessed using time-dependent ROC analysis. In the overall cohort, the 2-year 4-variable KFRE demonstrated good discriminative ability, with a time-dependent AUC of 0.82 (95% CI: 0.73–0.91) (Fig. 1A).

Age-stratified analyses showed consistent discrimination across age groups. In patients aged ≥65 years, the AUC remained robust, whereas slightly higher discriminative performance was observed in patients aged <65 years (Fig. 1B and C). Overall, these findings indicate preserved predictive accuracy of the KFRE model irrespective of age.

### Calibration performance at 2 years

**Figure 2:**
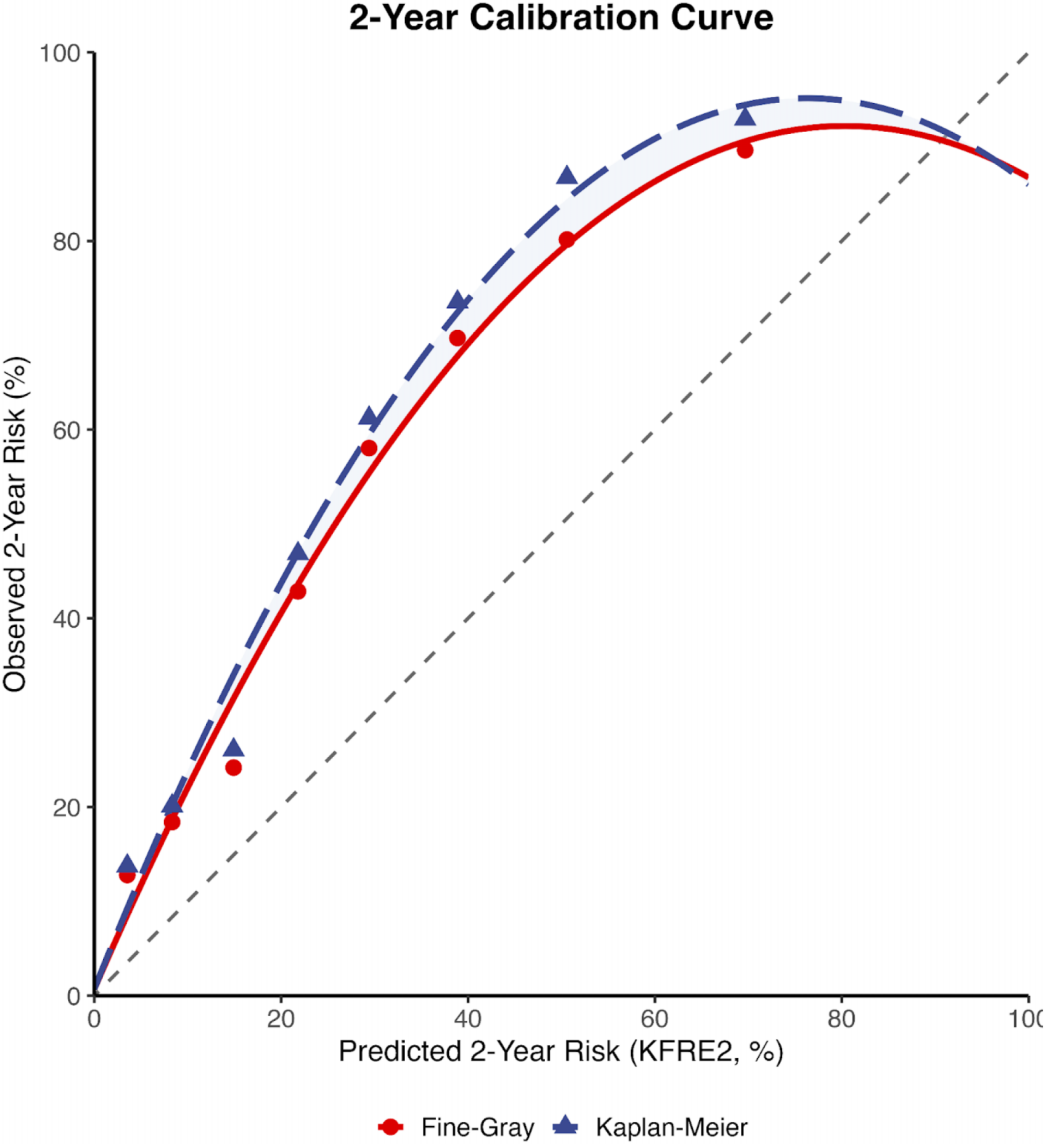
Calibration plot of the KFRE model at 2 years. Observed cumulative incidence of KRT across predicted risk deciles, estimated using Fine–Gray competing risk regression.

Calibration of the four-variable KFRE at 2 years was assessed using decile-based plots comparing predicted risk with observed cumulative incidence of KRT, estimated using Fine–Gray competing risk regression. The calibration plots showed a progressive increase in observed cumulative incidence across increasing risk deciles, indicating appropriate risk ordering, with mild underestimation of absolute risk (Fig. 2).

CITL analysis demonstrated mild underestimation of absolute risk. Negative CITL values indicate systematic underestimation of absolute risk. CITL was −0.21 (95% CI: −0.26 to −0.13) using the Fine–Gray estimator and −0.24 (95% CI: −0.28 to −0.17) using Kaplan–Meier estimation. The Brier score at 2 years was 0.23, reflecting acceptable overall predictive accuracy (Table 2).

**Table 2:** Quantitative calibration metrics of the KFRE model at 2 years. CITL and Brier score were used to assess calibration of the four-variable KFRE for prediction of KRT initiation at 2 years.

Metrics	Estimate (95% CI)
CITL (Kaplan–Meier)	−0.2408 (−0.2807 to −0.1735)
CITL (Fine–Gray)	−0.2091 (−0.2602 to −0.1314)
Brier score	0.2340 (0.2057–0.2622)

0 indicates perfect calibration, −1 indicates complete understimation, and 1 indicates complete overestimation.

### Risk stratification and competing risk analysis in patients aged ≥65 years

To evaluate the clinical relevance of KFRE-based risk stratification in older patients, cumulative incidence of KRT initiation at 2 years was analyzed in the subgroup aged ≥65, stratified according to a KFRE threshold of 20%.

**Figure 3:**
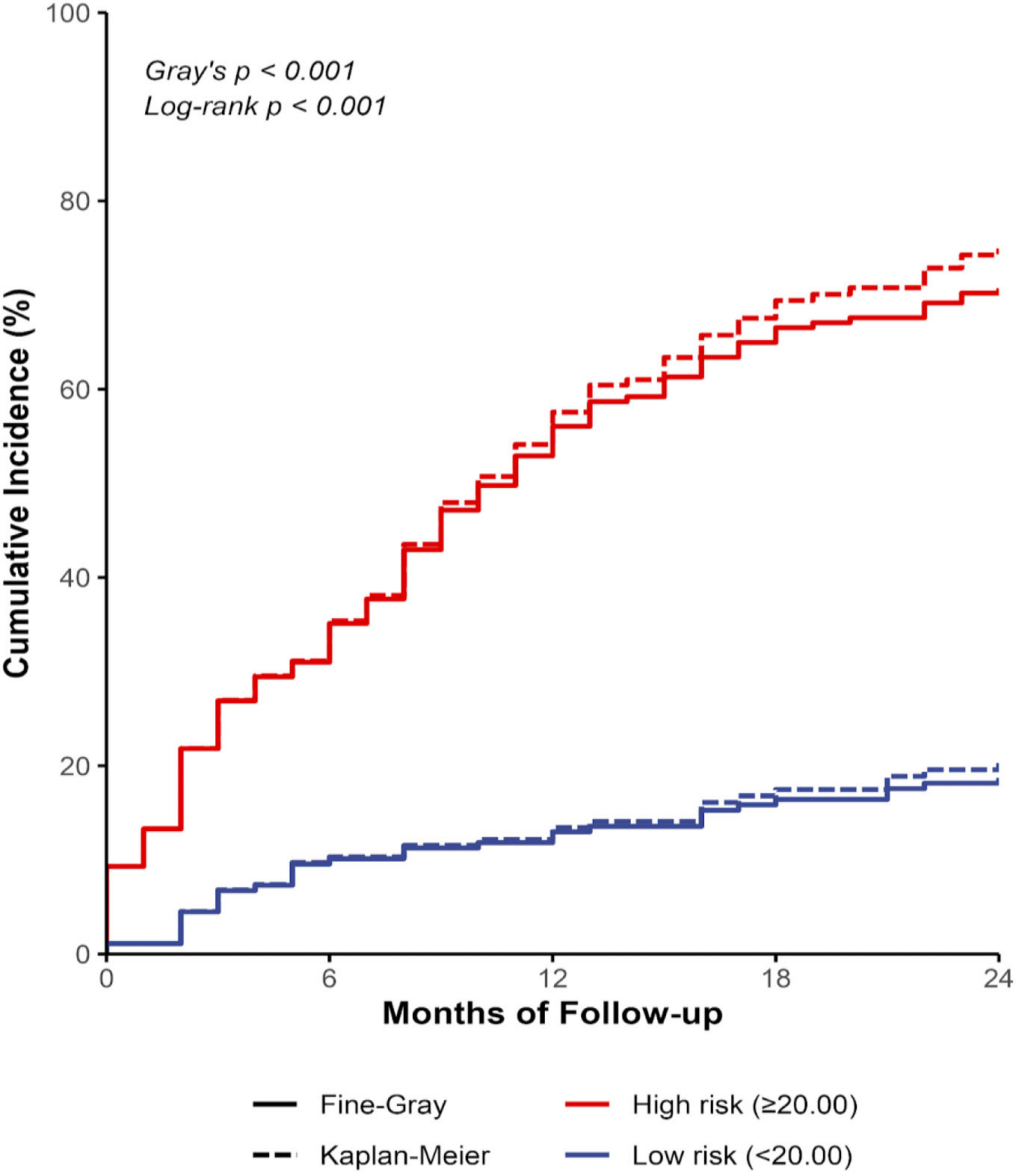
Cumulative incidence of KRT initiation at 2 years in patients aged ≥65 years, stratified by KFRE risk category (≥20% vs <20%). Comparison of Fine–Gray (Gray’s test) and Kaplan–Meier (log-rank test) estimators stratified by KFRE risk group (≥20% vs <20%). For the Fine–Gray model, the SHR was 5.921 (95% CI: 4.079–8.596; *P* < .001). For the Cox/Kaplan–Meier model, the cause-specific hazard ratio was 6.271 (95% CI: 4.311–9.124; *P* < .001).

A clear separation between risk groups was observed (Fig. 3). In Fine–Gray competing risk regression adjusted for age and sex, a KFRE ≥20% was associated with a significantly higher risk of initiating KRT (SHR 5.92; 95% CI: 4.08–8.60; *P* < .001). Consistent results were obtained using cause-specific Cox regression, with a hazard ratio of 6.27 (95% CI: 4.31–9.12; *P* < .001). Both Gray’s test and the log-rank test confirmed statistically significant differences between risk strata. Detailed hazard ratio estimates are provided in Supplementary Table S2.

### Clinical utility assessment by decision curve analysis

**Figure 4:**
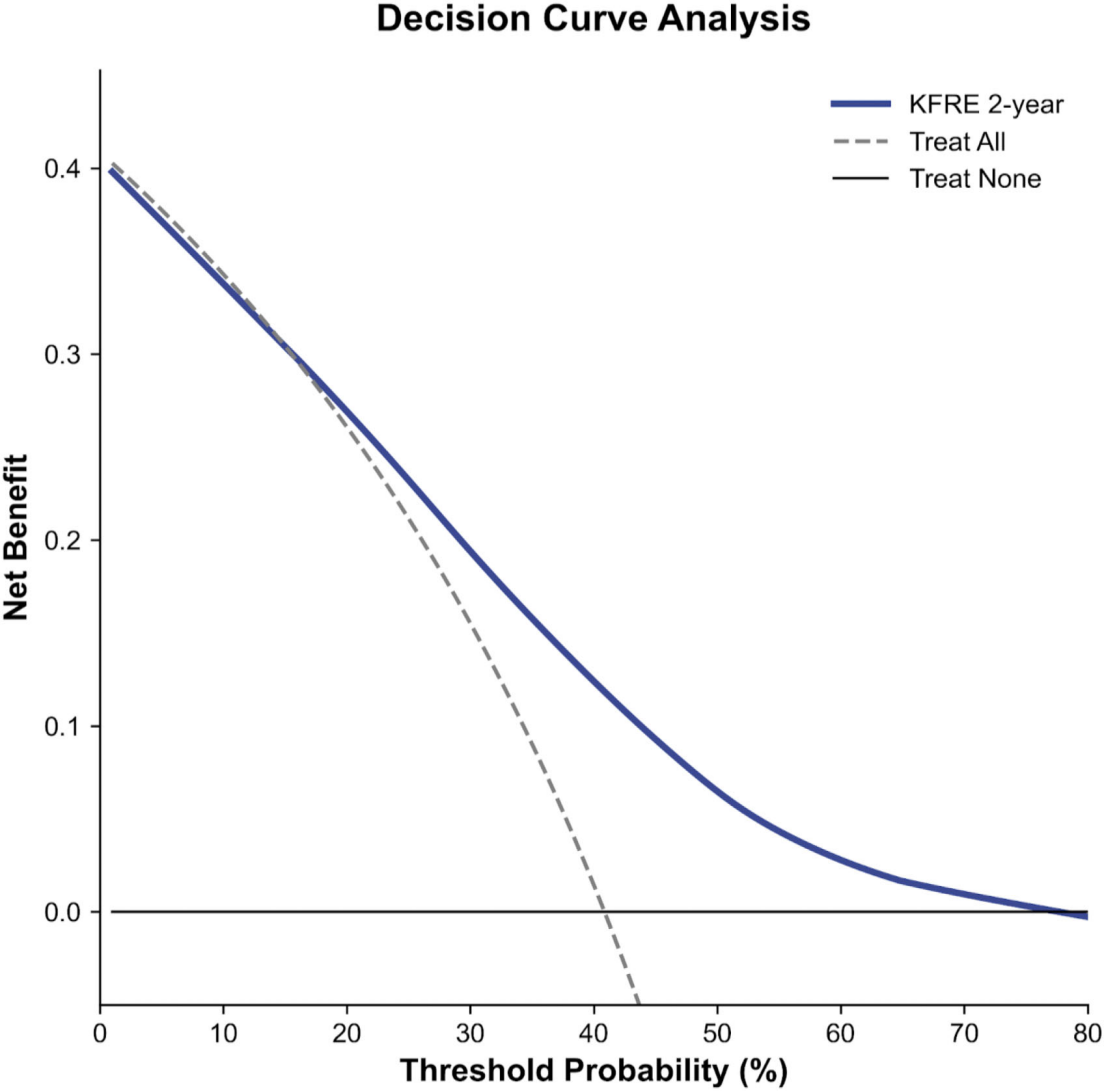
DCA of the KFRE model at 2 years. Net clinical benefit across a range of threshold probabilities for KRT initiation compared with “treat-all” and “treat-none” strategies.

The clinical usefulness of the KFRE model was further assessed using DCA. Across a range of clinically relevant threshold probabilities for KRT initiation (∼5%–25%), the four-variable KFRE demonstrated a positive net clinical benefit compared with both “treat-all” and “treat-none” strategies (Fig. 4).

These findings support the potential value of the KFRE model in guiding clinical decision-making related to referral, monitoring intensity, and preparation for KRT in patients with ACKD.

### Subgroup analysis by age (≥65 vs <65 years)

In predefined subgroup analyses, the KFRE model showed comparable discriminative performance in patients aged ≥65 years and <65 years. Despite the higher burden of competing mortality risk in older patients, KFRE-based risk stratification remained informative, supporting its applicability across age groups. Baseline characteristics differed between age strata (Supplementary Table S1). Patients aged ≥65 years had a lower UACR and lower serum creatinine, a higher burden of vascular aetiology and comorbidities, and a higher competing risk of death, whereas younger patients more frequently had glomerular disease and higher albuminuria.

## DISCUSSION

In this retrospective cohort of patients with ACKD and baseline eGFR <20 ml/min/1.73 m^2^, the 2-year 4-variable KFRE demonstrated good discriminative performance and acceptable calibration despite competing mortality. These findings were consistent across age strata, including in patients aged ≥65 years, a group characterized by a higher incidence of death before KRT initiation when compared to younger patients [3, 4].

The discriminative performance observed in our study (time-dependent AUC 0.82) is in line with previous international and Iberian validations of the KFRE in ACKD populations [7, 9, 13–15]. As reported in earlier studies, the model preserved its ability to rank patients according to short-term risk of kidney failure, even in elderly individuals [9, 13]. Our results confirm these findings within a multicenter Spanish cohort reflecting routine clinical practice.

A relevant aspect now is the explicit consideration of death as a competing event. In our population, 18.7% of patients died before initiating KRT within 2 years. In this context, competing risk methodology allows estimation of cumulative incidence that reflects the observed clinical course more accurately than approaches that treat death as noninformative censoring. Calibration analysis using Fine–Gray models [11] demonstrated appropriate risk ordering across predicted deciles, with mild underestimation of absolute risk. As emphasized in methodological guidance on external validation of prognostic models, accounting for competing events is particularly important in populations with substantial mortality risk [18].

Calibration remains an essential component of model evaluation. Although discrimination of the KFRE has consistently been high across settings [6] [7], absolute risk estimates may vary depending on baseline risk and healthcare context [19, 20]. In our hands, CITL showed mild underestimation, without marked systematic overprediction. This degree of miscalibration is comparable to that described in previous European validations [9, 14, 15].

The observed calibration pattern, with mild underestimation of risk particularly in lower predicted risk strata, may be explained by several factors. First, our cohort represents a selected population of patients with ACKD receiving nephrology care. This group has a higher baseline risk of progression than the broader CKD populations used in model development, which may contribute to systematic underestimation of absolute risk. Second, the smaller number of events in the lowest risk groups may increase the variability of the observed risk estimates and lead to greater apparent discrepancies in the calibration plots. Third, differences between the Kaplan–Meier and Fine–Gray estimates reflect the impact of competing risks, Kaplan–Meier tends to overestimate cumulative incidence, whereas Fine–Gray provides a more conservative and clinically relevant estimate in this context. Overall, these findings highlight the importance of interpreting absolute risk estimates within the clinical context and population characteristics.

In patients aged ≥65 years, a 2-year KFRE threshold of ≥20% identified a subgroup with substantially higher incidence of KRT initiation. The strong association observed in competing risk models suggests that short-term KFRE estimates may help identify individuals at higher probability of progression within a clinically relevant timeframe. Interpretation of these thresholds should remain within the broader clinical context, particularly in older patients with competing risks and heterogeneous disease trajectories [3, 4]. Current KDIGO guidelines recommend the use of validated risk prediction equations to complement eGFR in the evaluation and management of CKD [8]. Our findings are consistent with this risk-based approach and provide additional data from a Southern European multicenter cohort of patients already followed in ACKD clinics.

Beyond its prognostic performance, the clinical value of KFRE lies in its potential to support the organization of ACKD care. Risk-based stratification may help tailor the intensity of follow-up, prioritize patient education, and better time of preparation for KRT, including vascular or peritoneal access planning and pre-emptive transplant evaluation. In this regard, recent implementation data from a predialysis program suggested that systematic use of KFRE was associated with fewer late dialysis starts, lower use of temporary central venous catheters, and more timely preparation of dialysis access, supporting its potential role as a practical tool to improve predialysis management in routine nephrology care [22].

It is important to note that our cohort which was derived from nephrology clinics had a 2.5–3× higher risk of kidney failure than mortality. In these patients, KFRE shows mild underestimation of risk rather than overestimation as described in previous reports using alternative prediction model (KDPREDICT Ravani et BMJ). These previous studies were conducted in populations where death was 10 times more likely than kidney failure and therefore represent patients who are very unlikely to be managed by nephrologists or considered for dialysis access placement. We believe that calibration is largely dependent on the validation population rather than the choice of statistical method. As such, both the Kaplan–Meier and Fine and Gray methods show similar results in this study whereas a competing risk method may appear superior in a cohort where death is 10-fold more likely than kidney failure. For nephrologists who are providing care for patients with ACKD, models built in nephrology cohorts, such as KFRE, are likely better calibrated and more appropriate.

Strengths of this study include its multicenter validation as well as the inclusion of a population that is actually considered for access placement (ACKD under nephrology care). We also compared both conventional KM methods as well as competing risk methods (Fine and Gray) to assess calibration and found similar results. Several limitations should be acknowledged. The retrospective design may limit generalizability. Differences in event rates between participating centers likely reflect variation in case mix or clinical practice patterns and may influence absolute risk estimates. In our analysis, model performance was explored separately by center, including discrimination, cumulative incidence, calibration plots, and DCA. Discriminative ability remained within an acceptable range across centers, and KFRE-based risk stratification consistently identified groups with different risks of KRT. However, some variability in calibration was observed, likely reflecting differences in the baseline risk and patient characteristics between cohorts (Supplementary Figure S1). Follow-up was limited to 2 years, precluding assessment of longer-term performance, and other versions of the KFRE (e.g. UK version) were not tested. Finally, formal recalibration of the model was not performed.

In conclusion, in this real-world Spanish multicenter cohort of patients with ACKD, the 2-year 4-variable KFRE showed good discrimination, acceptable calibration with or without competing-risk methodology, and consistent performance across age groups. These findings support its use as a complementary tool for short-term risk stratification in ACKD care.

## Supplementary Material

sfag192_Supplemental_File

## Data Availability

The data underlying this article will be shared on reasonable request to the corresponding author.
