# Supplementary material for: Planning of kidney replacement therapy in advanced CKD using the KFRE formula in a Spanish multicenter cohort
Source: Clin Kidney J. 2026 Jun 9;19(7):sfag192. doi: 10.1093/ckj/sfag192 (PMC13366082; doi:10.1093/ckj/sfag192)
Supplement: sfag192_Supplemental_File [file sfag192_supplemental_file.pptx]

## Slide 1
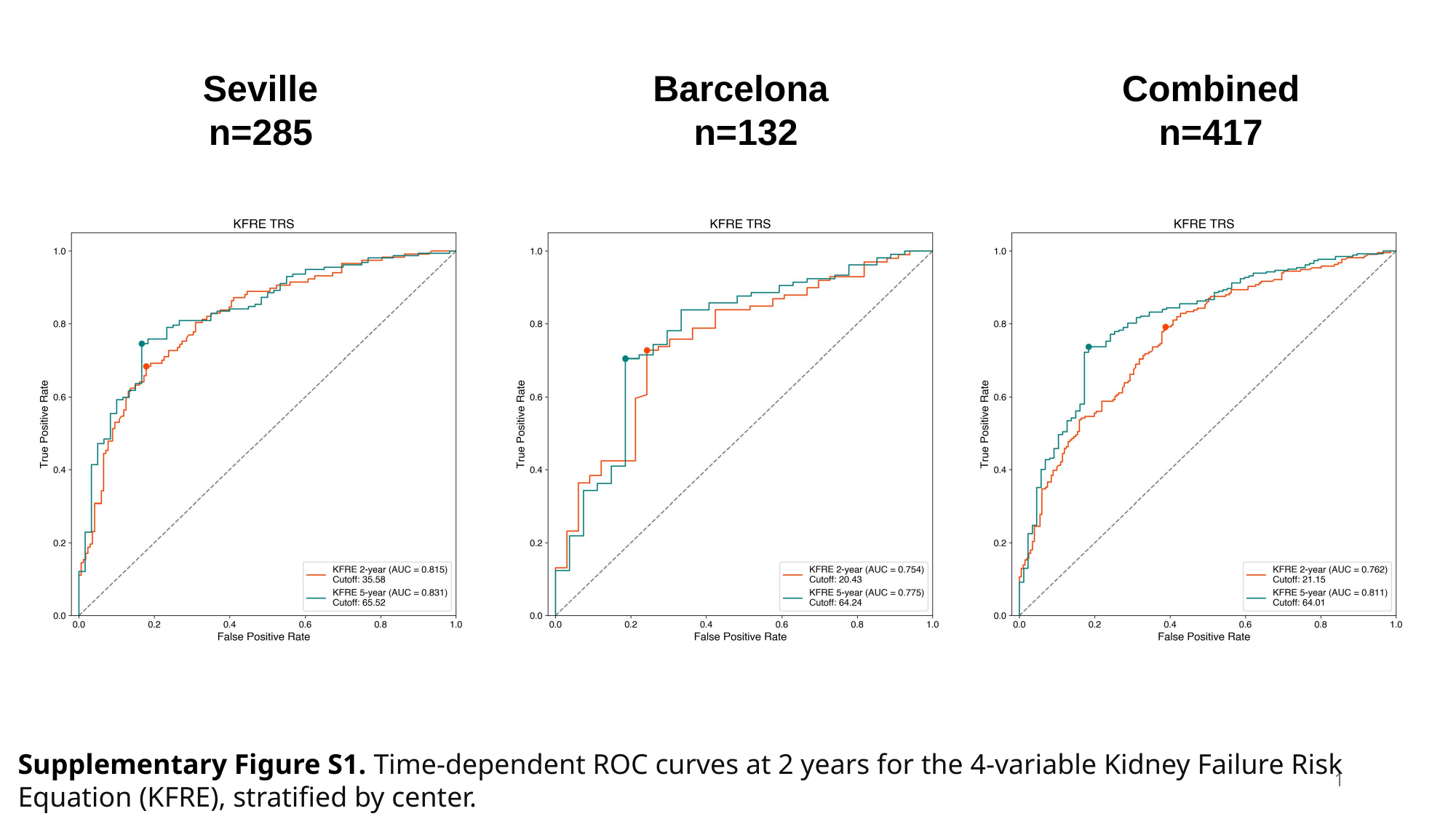

Seville
n=285
Barcelona
 n=132
Combined
n=417
Supplementary Figure S1. Time-dependent ROC curves at 2 years for the 4-variable Kidney Failure Risk Equation (KFRE), stratified by center.
1
